# Physiological and biochemical mechanisms underlying the role of anthocyanin in acquired tolerance to salt stress in peanut (*Arachis hypogaea* L.)

**DOI:** 10.3389/fpls.2024.1368260

**Published:** 2024-03-11

**Authors:** Guanghui Li, Xin Guo, Yanbin Sun, Sunil S. Gangurde, Kun Zhang, Fubin Weng, Guanghao Wang, Huan Zhang, Aiqin Li, Xingjun Wang, Chuanzhi Zhao

**Affiliations:** ^1^ Shandong International Joint Laboratory of Agricultural Germplasm Resources Innovation, Institute of Crop Germplasm Resources (Institute of Biotechnology), Shandong Academy of Agricultural Sciences, Jinan, China; ^2^ Center of Excellence in Genomics & Systems Biology (CEGSB), International Crops Research Institute for the Semi-Arid Tropics (ICRISAT), Hyderabad, India; ^3^ College of Agronomy, Shandong Agricultural University, Taian, China; ^4^ College of Life Sciences, Shandong Normal University, Jinan, China

**Keywords:** peanut, testa color, salt stress, anthocyanin, oxidation resistance, photosynthesis

## Abstract

Anthocyanin is an important pigment that prevents oxidative stress and mediates adaptation of plants to salt stress. Peanuts with dark red and black testa are rich in anthocyanin. However, correlation between salt tolerance and anthocyanin content in black and dark red testa peanuts is unknown. In this study, three peanut cultivars namely YZ9102 (pink testa), JHR1 (red testa) and JHB1 (black testa) were subjected to sodium chloride (NaCl) stress. The plant growth, ion uptake, anthocyanin accumulation, oxidation resistance and photosynthetic traits were comparatively analyzed. We observed that the plant height, leaf area and biomass under salt stress was highly inhibited in pink color testa (YZ9102) as compare to black color testa (JHB1). JHB1, a black testa colored peanut was identified as the most salt-tolerance cultivar, followed by red (JHR1) and pink(YZ9102). During salt stress, JHB1 exhibited significantly higher levels of anthocyanin and flavonoid accumulation compared to JHR1 and YZ9102, along with increased relative activities of antioxidant protection and photosynthetic efficiency. However, the K^+^/Na^+^ and Ca^2+^/Na^+^ were consistently decreased among three cultivars under salt stress, suggesting that the salt tolerance of black testa peanut may not be related to ion absorption. Therefore, we predicted that salt tolerance of JHB1 may be attributed to the accumulation of the anthocyanin and flavonoids, which activated antioxidant protection against the oxidative damage to maintain the higher photosynthetic efficiency and plant growth. These findings will be useful for improving salt tolerance of peanuts.

## 1 Introduction

Peanut (*Arachis hypogaea* L.) is widely cultivated in more than 100 countries, with the annual yield of 43.98 million tons, representing one of the most important oil and cash crops specially in Africa and Asia (Zhao et al., 2020). Peanut seeds containing 44% to 56% oil, are the fourth largest source of edible oil globally. In addition, peanuts are rich in protein (22%-30%), carbohydrates (10%-20%), as well as vitamins, essential fatty acids, and necessary minerals for human nutrition (Mondal et al., 2020). China has the greatest peanut production in the world, and is second, after India in the plant area (4.45 million hectares). China is expected to account for more than 40% of the world’s peanut oil (Zhao et al., 2012). Over the past few decades, the most planting and processing of peanut cultivars are with pink or red testa. The variation of peanut testa color is mainly due to the difference in anthocyanin content, a highly diverse group of secondary metabolism product that contribute to plants color. Recently, the peanut planting and consumption pattern with market demands has been changed. Peanuts with purple and black testa color have attracted increasing attention of consumers in the market, due to their higher anthocyanin and microelements contents important for human health (Bonku and Yu, 2020).

Soil salinization is a major threat for agriculture worldwide, limits crop yield and restricts use of arable and uncultivated land. It has been estimated that more than 8% of the world’s land is affected by salinity, and it is continuously increasing (Ait-El-Mokhtar et al., 2020). Soil salinity causes a more than 20% reduction of agricultural yields (Porcel et al., 2012). Salinity can cause osmotic stress, ion imbalance and oxidative damage to plants normal physiological processes, and the plants have evolved sophisticated mechanisms to cope with salinity stress (Abogadallah, 2010).

Peanut was considered to be moderately salt-tolerant crop by the Food and Agriculture Organization (FAO). However, peanut production suffers great challenge by salt-stress because of the widely distributed saline-alkaline land in major peanut regions in China, India and United States of America (Luo et al., 2021). Salinity significantly inhibits peanut germination, relative growth rate and dry mass production (Meena et al., 2016), induced photo-inhibition because of damaging the photosynthetic apparatus (Qin et al., 2011), and restrained Ca, K and other mineral elements uptake, and eventually reduced yield and quality (Zhang et al., 2020). Significant genetic diversity is available for salinity tolerance among peanut germplasms (Zou et al., 2020).

In the past few years, a series of agronomic measures were widely adopted to enhance salt-tolerance of peanut, including selecting salt-tolerant cultivars (Meena et al., 2016; Zhang et al., 2020), mulching film and potassium application (Chakraborty et al., 2016; Meena et al., 2022), inoculating salt-tolerant rhizobacteria and arbuscular mycorrhizal fungi (El-Akhal et al., 2012; Sharma et al., 2016; Qin et al., 2021), and exogenous growth regulator (Tian et al., 2019; Li W. J. et al., 2022). In addition, the identification and utilization of salt tolerant genes have been used to improve the salinity tolerance of peanut cultivars (Banavath et al., 2018; Zhu et al., 2021). However, the production and economical outcome of peanut in saline-alkali soil was still limited. Therefore, improvement of salt tolerance is necessary to minimize the resulting yield loss due to salt stress.

Secondary metabolites play a key role in the adaptation of plants to the changing environment and in overcoming stress conditions. Anthocyanin, a secondary metabolite in plants, serves as an important antioxidant, increases the antioxidant activity and enhances the ability of abiotic stress tolerance (Xu and Rothstein, 2018). The salt-tolerant cultivars showed higher anthocyanin content and total antioxidant activity than the salt-sensitive cultivars under salt stress, exhibited more physiological activities (Daiponmak et al., 2010). Accumulation of higher levels of anthocyanin is considered as one of the selection criteria for salt tolerance (Eryılmaz, 2006). The cultivars with deep red or black color (more intense anthocyanin) in apple (Wang et al., 2015), rice (Chunthaburee et al., 2016), spinach (Kitayama et al., 2019) and *Brassica napus* (Kim et al., 2017) suffered less physiology and cellular damages and lower growth inhibition under salt stress as compared to the ones with less anthocyanin content, showed a stronger salt tolerance.

Most of the previous studies on salt stress or enhancing salt-tolerance in peanut mainly used common cultivars with pink testa color. There is significant difference in anthocyanin content among peanut cultivars with different testa colors. However, there are no reports on the salt tolerance and regulatory mechanism of peanut cultivars with black and red testa colors. Therefore, a pot experiment was conducted in greenhouse to screen the salt tolerance among three peanut cultivars with different testa colors. The data recorded on anthocyanin and flavonoid contents, ion uptake, antioxidant activity and photosynthetic traits of three peanut cultivars in response to salinity stress. This work provided a foundation for screening salt resistant peanut cultivars and peanut high-yield cultivation in saline-alkali soil, and laid a basis for further revealing the salt-tolerant mechanism of colored peanut.

## 2 Materials and methods

### 2.1 Plant materials and treatments

**Figure 1:**
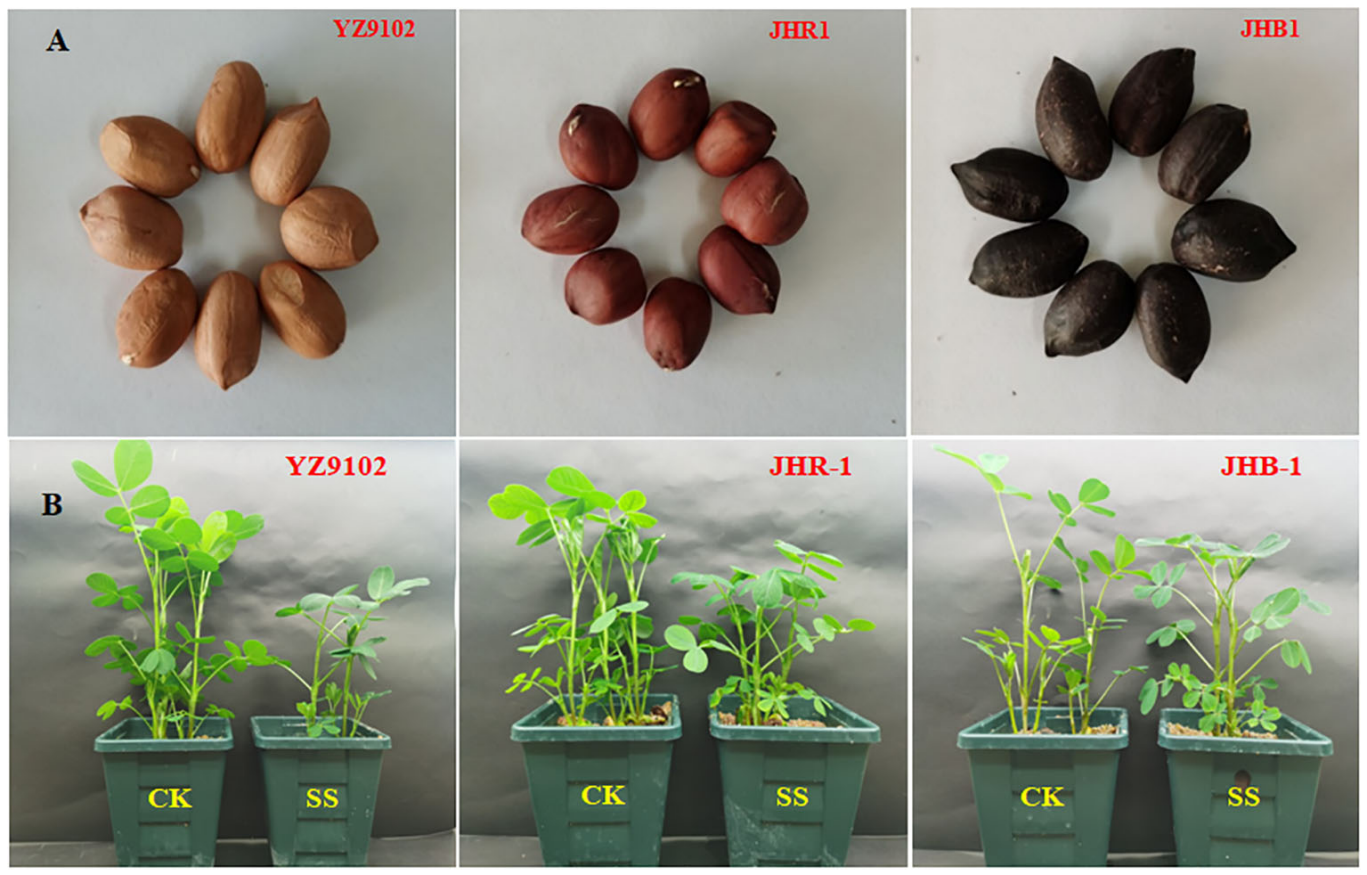
The seeds of three peanut cultivars **(A)** and the phenotype of three peanut cultivars under CK and SS **(B)**. CK, meaning plants were watered by Hoagland solution without NaCl, and SS, meaning plants were watered by Hoagland solution with 150 mM NaCl.

Three peanut cultivars, YZ9102 (pink testa), JHR1 (red testa) and JHB1 (black testa), were used in this study (**Figure 1A**). YZ9102, bred by Henan Academy of Agriculture Sciences, was offspring of “BS1016” (♀) × “A. *coacoense*” (♂). JHR1 and JHB1, bred by our group, were offspring of “YZ9102” (♀) × “ZH12” (♂), and “FH1” (♀) × “ZH9” (♂), respectively.

The pot experiment was conducted in a growth chamber in controlled conditions. Matured seeds no pests or diseases and with uniform size and weight were selected, and surface sterilized by soaking in 75% ethyl alcohol with slightly shaking for 5 min and rinsed using sterilized deionized water for 3 times. Three seeds were sown in each square plastic pot (10.5 cm top diameter, 11 cm height) filled with sterilized clean river sand 0.95 kg, and two uniform seedlings were kept in each pot after germination. The climatic chamber was controlled at 25°C, alternation of light and dark period at 16 h/8 h. Seven days after germination, the plants of each cultivar were divided into two groups. Our preliminary experiment showed that the salt tolerance had the most significant difference in ten peanut cultivars under 150 mM NaCl stress. So, one group was designated as control (CK) irrigated by the Hoagland solution, the other group was challenged with salt stress treatment (SS) irrigated by the Hoagland solution and salt concentration of 150 mM NaCl. Each treatment consisting of twenty pots was watered daily uniformly with 100 mL Hoagland solution with or without NaCl per pot to maintain the treatment level.

After 1, 5 and 10 days of salt stress, leaf materials were sampled and immediately transferred into liquid nitrogen and stored at -80°C to estimate enzymatic activity. The flavonoid and anthocyanin contents were determined with the leaf samples at 5 and 10 days of salt stress. Leaf gas exchange and chlorophyll fluorescence parameters were measured on the second top leaf on the main stem after 5 and 10 days of salt stress. K^+^, Na^+^, and Ca^2+^ contents in leaf and stem were determined after 10 days of salt stress. Furthermore, the plants were harvested at 10 d after salt stress for biomass and phenotypic analysis.

### 2.2 Determination of dry weight and leaf area

Five representative plants were selected from each treatment after 10 d of salt stress, and separated into roots, stems and leaves. In each treatment, fifty random leaflets were punched into wafers with 1.2 cm diameter avoiding the main vein. All plant samples were oven dried at 105°C for 30 min followed at 80 °C until reaching a constant dry weight. The total dry weight (DW), the total wafers area (S1), wafers dry weight (M1) and other leaves dry weight (M2) were recorded. The leaf area (S) was calculated using the following formula: S= S1× (M1+M2)/M1.

### 2.3 Determination of Na^+^, K^+^ and Ca^2+^ ions

Dried leaf and stem samples were milled to powder, weighed, and then digested by nitric acid in a bottle tube at 320°C for 5 h. Na^+^, K^+^ and Ca^2+^ ion concentration was measured by atomic adsorption spectrometer with a flame photometer (ZL5100, PerkinElmer Inc., USA). Each treatment was repeated with three biological replicates and three technical replicates.

### 2.4 Determination of total flavonoids

Total flavonoids content was measured by the colorimetric method (Jia et al., 1999). 0.3 g fresh leaf was cutinto pieces and extracted in a 60°C water bath for 1 hour in a test tube with 70% ethanol (10 mL), and then filtered through two layers of filter paper. The 5 mL filtrate was added into 0.5 mL of 5% NaNO_2_ and 6 min later 0.5 mL of 10% Al(NO_3_)_3_. After 6 min, 4 mL of 4% NaOH was added to the mixture. The solution was mixed well, and after 15 min, the absorbance was recorded at 510 nm in spectrophotometer (U-3000, HITACHI, Japan).

### 2.5 Determination of anthocyanin

The anthocyanin content was measured using the protocol reported by Zhang et al. (2022). Frozen leaf (approximately 50 mg) was ground in a 5 mL centrifuge tube using liquid nitrogen. Then, homogenized sample was extracted at 4°C by adding 700 μL acidic methanol (the volume ratio of methanol to HCl is 99:1). After overnight incubation, the homogenates were centrifuged at 4°C at 12,000×*g* for 10 min. About 600 μL supernatant was collected and mixed with 1 mL trichloromethane and 400 μl distilled water, centrifuged at 4°C at 12,000×*g* for 10 min. The absorbance of the supernatant was recorded at 530 and 657 nm using spectrophotometer (U-3000, HITACHI, Japan).

### 2.6 Antioxidant enzymes activity and MDA content

Leaf samples (0.5 g) were homogenized using a pre-cooled mortar in 50 mM potassium phosphate buffer (pH7.8) at 0~4°C. The homogenate was filtered through two layers of filter paper and centrifuged at 10,000×*g* for 20 min at 4°C. The supernatant was used for enzyme activity analysis.

Superoxide dismutase (SOD) activity was measured by the method of Giannopolitis and Ries (1977). The reaction mixture consisted of 50 mM phosphate buffer (pH 7.8), 13 mM methionine, 75 mM nitrotetrazolium blue chloride (NBT), 0.1 mM ethylene diamine tetraacetic acid (EDTA) and 2 mM riboflavin. Reactions with 50 µL enzyme extract and 3 mL reaction mixture were carried out in a light incubator under a light intensity of 4000 Lux for 30 min. One unit of SOD was defined as the amount of enzyme which causes 50% inhibition of the NBT reduction. The reduction of NBT was measured by an ultraviolet spectrophotometer at 560 nm.

Peroxidase (POD) activity was determined based on guaiacol colorimetric method. Reaction mixture contained 50 mL 100 mM potassium phosphate (pH 6.0), 30 µL 0.3 mM guaiacol, and 20 µL 30% H_2_O_2_. The 20 μL enzyme solution and 3 mL reaction mixture were added into the colorimetric cup to start the reaction. Absorbance was recorded at 470 nm at every 30 s intervals for a total of 5 readings. The activity of the POD enzyme was expressed by the change of value of absorbance per minute (Nickel and Cunningham, 1969).

Catalase (CAT) activity was measured according to Aebi (1984). 50 μL enzyme solution was added into 3 mL reaction system (2.4 mL of 100 mM potassium phosphate (pH 7.0), 0.6 mL of 100 mM H_2_O_2_). Absorbance was recorded at 240 nm at every 30 s intervals for a total of 5 readings. The activity of the CAT enzyme was expressed by the reduction of absorbance per minute.

Malondialdehyde (MDA) was assayed by the thiobarbituric acid reaction method (Hodges et al., 1999). Frozen sample of 0.5 g was homogenized in 0.1% (w/v) trichloroacetic acid (TCA) solution. The homogenate was centrifuged at 12,000×*g* for 10 min. 1 mL supernatant was added to 2 mL of 20% TCA containing 0.6% thiobarbituric acid (TBA) in a clean glass tube. The mixture was heated in a water bath at 90°C for 30 min, cooled on ice immediately, and centrifuged at 4000×*g* for 10 min. The absorbance was recorded at 600, 532 and 450 nm.

### 2.7 Determination of gas exchange

After 5 and 10 days of salt stress, leaf photosynthesis was determined on the second top leaf on the main stem of five plants in each treatment. Leaf photosynthesis rate (*P*_n_), transpiration rate (*T*_r_) and stomatal conductance (*G*_s_) were measured with a portable photosynthesis system (Li-6400; LI-COR Inc., Lincoln, NE, USA) at 9:00-11:30 AM. The chamber was equipped with a red/blue LED light source setting PAR at 1200 µmol m^-2^ s^-1^ and ambient atmospheric CO_2_ levels at 385μmol mol^-1^. The water use efficiency of leaves (WUE) was calculated by using formula *P*_n_/*T*_r_.

### 2.8 Measurement of leaf chlorophyll fluorescence

Chlorophyll fluorescence parameters were measured with a portable pulse modulated fluorometer (FMS-2, Hansatech, England) on the same leaf whose gas exchange was measured. Tested leaves were kept in the dark for 30 min before measurement. The minimum (*F*_0_), maximum fluorescence (*F*_m_), steady state fluorescence (*F*_s_), minimum fluorescence (*F*_0_’) and maximum fluorescencein the irradiation-adapted state (*F*_m_’) were determined. Quantum yield of PS II (*ΦPSII*), maximal photochemical efficiency (*F*_v_/*F*_m_) photochemical quenching coefficient (*qP*), electron transport rate (*ETR*), and non-photochemical quenching coefficient (*qNP*) were calculated as described by Rasouli et al. (2023).

### 2.9 Statistical analysis

All parameters were measured in at least three replications and expressed as means± standard deviation. The average of each trait was calculated with Microsoft Excel 2010, plotted by Sigmaplot10.0. Duncan’s multiple range test was used to determine the significant difference between treatments (*P*<0.05) by SPSS Statistics 23. The relative values were calculated via the trait values under salt stress compared with those of CK.

## 3 Results

### 3.1 Plant growth and biomass accumulation

All of the tested peanut cultivars were observed to certain growth inhibition when subject to salt stress (**Table 1** and **Figure 1B**). The plant stem height, leaf areas, fresh weight of all cultivars were significantly decreased as compared to CK under salt stress, those relative values were 0.60, 0.48 and 0.72 in YZ9102, 0.59, 0.52 and 0.79 in JHR1, and 0.69, 0.67 and 0.92 in JHB1, respectively. The relative total dry weight values of YZ9102, JHR1 and JHB1 were 0.91, 0.93 and 0.95 under salt stress as compared to CK, reflecting the different salt tolerance of three cultivars.

**Table 1:** The phenotype and individual biomass accumulation of three peanut cultivars after 10 days under salt stress.

Cultivars	Treatment	Plant height (cm)	Leaf area (cm^2^)	Fresh weight (g)	Dry weight (g)
YZ9102	CK	11.80 ± 0.57a	92.70 ± 5.84a	7.50 ± 0.38a	0.78 ± 0.04a
	SS	7.04 ± 0.60b	44.19 ± 5.27b	5.38 ± 0.49b	0.71 ± 0.07a
JHR1	CK	10.96 ± 0.71a	76.71 ± 9.64a	5.26 ± 0.19a	0.57 ± 0.02a
	SS	6.44 ± 0.59b	39.91 ± 5.64b	4.15 ± 0.21b	0.53 ± 0.05a
JHB1	CK	10.80 ± 0.81a	77.08 ± 7.66a	5.54 ± 0.23a	0.79 ± 0.02a
	SS	7.50 ± 0.69b	51.54 ± 3.68b	5.12 ± 0.11a	0.75 ± 0.04a

Data are means ± SD of three biological replicates. Different lowercase letters indicate significantly different between CK and SS at p<0.05. CK, meaning plants were watered by Hoagland solution without NaCl, and SS, meaning plants were watered by Hoagland solution with 150 mM NaCl.

### 3.2 Content of Na^+^, K^+^ and Ca^2+^

**Figure 2:**
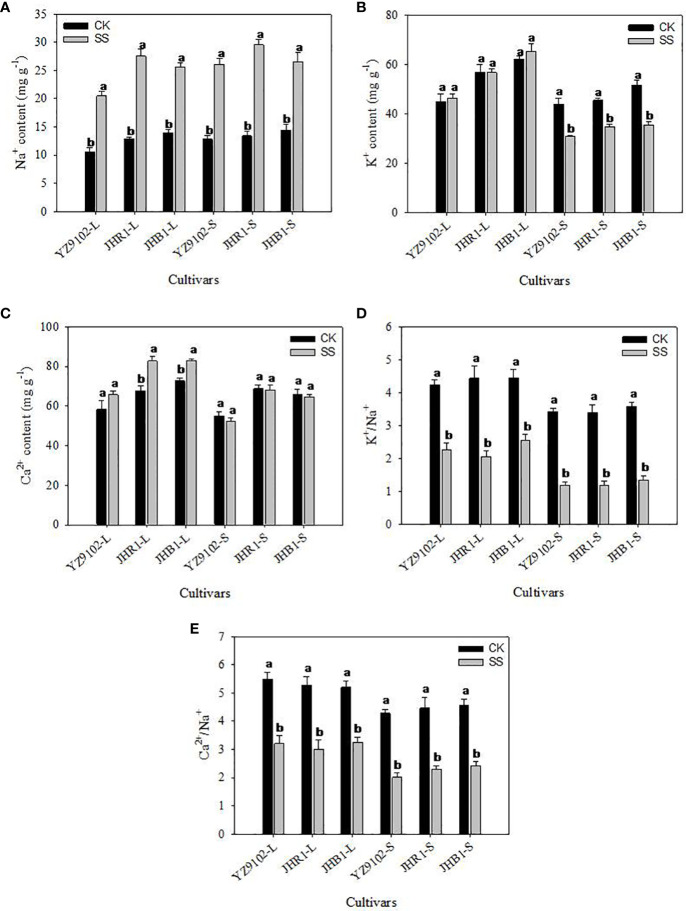
The Na^+^ **(A)**, K^+^ **(B)**, Ca^2+^ **(C)**, K^+^/Na^+^ **(D)** and Ca^2+^/Na^+^ **(E)** of three peanut cultivars in leaves and stems under salt stress for 10 days. L and S represented leaf and stem, respectively. Different lowercase letters in the bar graph indicate significantly different between CK and SS at *p*<0.05. CK, meaning plants were watered by Hoagland solution without NaCl, and SS, meaning plants were watered by Hoagland solution with 150 mM NaCl.

Salt stress significantly enhanced the content of Na^+^ in leaf and stem as compared to CK in all three cultivars (**Figure 2A**). The Na^+^content in JHB1 showed the minimum increase of 83.10% and 84.44% in leaf and stem, and the maximum increase of 114.50% in leaf in JHR1, 103.71% in stem in YZ9102. With or without salt stress, the K^+^ content of JHB1 was the most, following by JHR1.To compare with CK, all cultivars exhibited a slightly increased K^+^ content in the leaf, however, a significantly decreased K^+^ in the stem under salt stress (**Figure 2B**). The content of K^+^ was slightly declined (23.65%) in stem of JHR1, and approximately 30% reduction in the other two cultivars under salt stress. With or without salt stress, JHR1 and JHB1 showed similar content of Ca^2+^, and significantly higher than YZ9102. Salt stress significantly increased Ca^2+^content of Ca^2+^ in the leaf tissue of three cultivars, and inappreciably restrained the content in stem as compared to CK (**Figure 2C**). The maximum increase (22.01%) of Ca^2+^ content in leaf was found in JHR1, followed by JHB1 and YZ9102.

The values of K^+^/Na^+^ were approximately consistent among all three cultivars, whereas, dramatically decrease under salt stress, as compared to CK (**Figure 2D**). K^+^/Na^+^ in leaf were decreased by 46.44%, 53.58% and 42.61% in YZ9102, JHR1 and JHB1, respectively. While, in stem the K^+^/Na^+^ were reduced by 65.44%, 65.39% and 62.74% among YZ9102, JHR1 and JHB1 cultivars respectively under salt stress. The values of Ca^2+^/Na^+^ showed the similar variation trend with K^+^/Na^+^ among the three cultivars under salt stress, whereas those experienced a smaller reduction under salt stress than that of K^+^/Na^+^ (**Figure 2E**).

### 3.3 Content of flavonoid and anthocyanin

**Figure 3:**
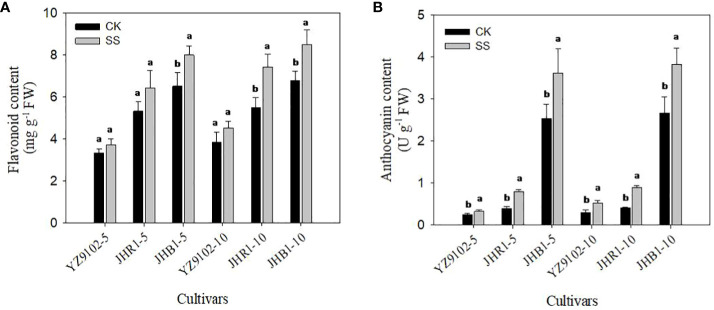
The contents of flavonoid **(A)** and anthocyanin **(B)** in leaves under salt stress for 5 and 10 days. Different lowercase letters in the bar graph indicate significantly different between CK and SS at *p*<0.05. CK, meaning plants were watered by Hoagland solution without NaCl, and SS, meaning plants were watered by Hoagland solution with 150 mM NaCl. The 5 and 10 in abscissa represented 5 and 10 days after salt stress, respectively.

The flavonoid content of YZ9102, JHR1 and JHB1 increased by 11.45%, 21.18% and 22.80% after 5 days of salt stress as compare to CK, further at 10 days of salt stress it was increased by 17.26%, 35.13% and 25.43% (**Figure 3A**). The higher level of flavonoid content was observed in JHB1, followed by JHR1 and YZ9102 in both control and salt stress. Salt stress significantly increased anthocyanin content in all three cultivars as compared to CK (**Figure 3B**). Maximum increase in anthocyanin content was recorded in JHR1, increasing 103.17% and 117.99% in contrast to CK after 5 and 10 days of salt stress, respectively. In cultivar YZ9102, the trend of increased anthocyanin content was much more apparent at 10 day of salt stress (74.39%) than that at 5 days (32.51%). Whereas, it was stable in cultivar JHB1 increased by 42.37% and 43.46% at 5 and 10 days of salt stress. With or without salt stress, the content of anthocyanin in JHB1 was significantly higher than those in JHR1 and YZ9102.

### 3.4 Antioxidant enzyme activity

**Figure 4:**
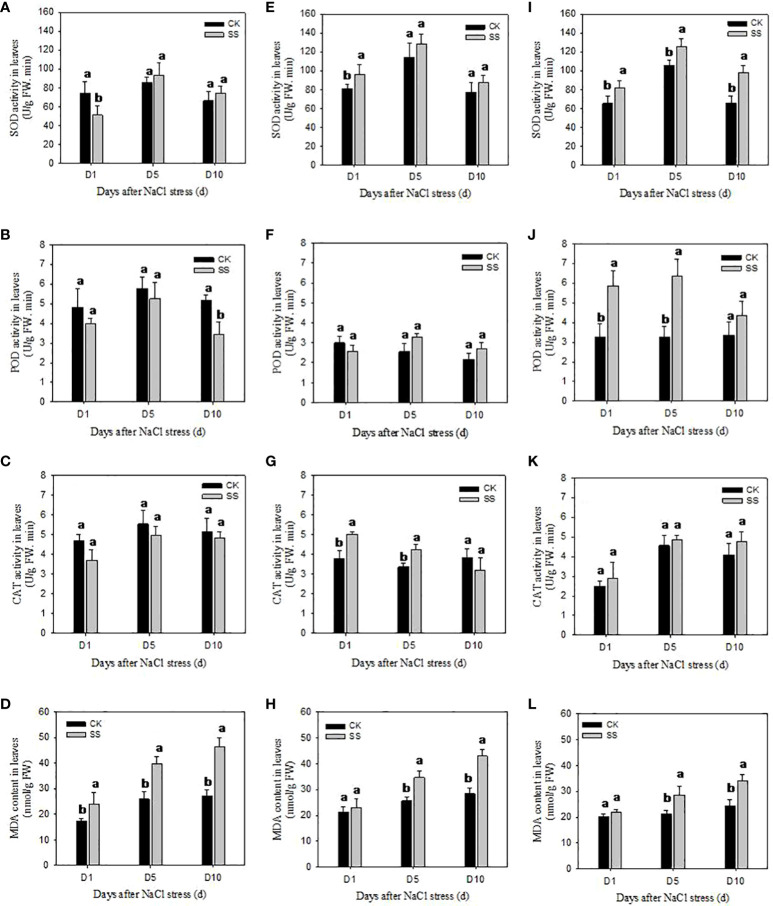
The antioxidant enzyme activities and MDA content in leaves of YZ9102 **(A–D)**, JHR1 **(E–H)** and JHB1 **(I–L)** under salt stress. D1, D5, D10 represent salt stress for 1, 5 and 10 days. Different lowercase letters in the bar graph indicate significantly different between CK and SS at *p*<0.05. CK, meaning plants were watered by Hoagland solution without NaCl, and SS, meaning plants were watered by Hoagland solution with 150 mM NaCl.

We observed significant differences among the responses of antioxidant enzyme activities of SOD, POD and CAT in three cultivars to salt stress (**Figure 4**). In comparison with CK, the SOD activity of YZ9102 was significantly decreased by 30.80% after 1 day of salt stress, but it was a slightly increased at 5 and 10 days of salt stress. There was a relatively constant increase in SOD activity of JHR1 during 10 days of salt stress. Significant increase in SOD activity in JHB1 was observed under salt stress, and it showed maximum increase (49.43%) at 10 day of salt stress. The increase of SOD activity in JHB1 was higher than that in JHR1 and YZ9102.

POD activity was significantly decreased in YZ9102 under salt stress as compared to CK. The maximum decrease (34.01%) was recorded at 10 days of salt stress. POD activity of cultivar JHR1 was decreased by 13.77% at 1 day of salt stress, however, increased by 27.72% and 23.38% at 5 and 10 days of salt stress. POD activity of JHB1 was significantly increased under salt stress, and the maximum increase (94.80%) was observed at 5 days of salt stress. JHB1 showed higher levels of POD activity than the other cultivars.

The response pattern of CAT activity of YZ9102 under salt stress was similar with that of its POD activity. The CAT activity of JHR1 significantly increased by 32.96% and 25.11% after 1 and 5 days of salt stress as compared to CK, however it was decreased by 16.92% at 10 days of salt stress. Though the CAT activity of JHB1 was slightly increased under salt stress, there was no significant difference between salt stress and CK.

MDA content was significantly increased in all three cultivars under salt stress (**Figure 4**). The MDA content of YZ9102 was recorded higher as compared to other two cultivars, and it showed a rapid increase from 37.00% to 72.05% from 1 to 10 days as compared to CK under salt stress. The MDA content of JHR1 and JHB1 exhibited a slight increase at 1 day of salt stress, however it was increased by 51.56% and 39.17% at 10 days of salt stress as compared to CK, respectively. JHB1 showed the minimum MDA content in the presence or absence of salt stress.

### 3.5 Photosynthetic parameters

**Figure 5:**
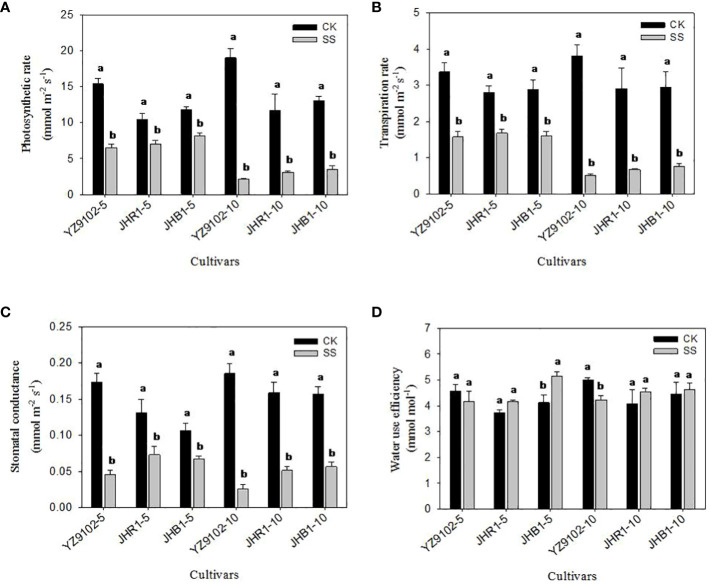
The photosynthetic rate **(A)**, transpiration rate **(B)**, stomatal conductance **(C)** and water use efficiency **(D)** of peanut leaves under salt stress for 5 and 10 days. Different lowercase letters in the bar graph indicate significantly different between CK and SS at *p*<0.05. CK, meaning plants were watered by Hoagland solution without NaCl, and SS, meaning plants were watered by Hoagland solution with 150 mM NaCl. The 5 and 10 in abscissa represented 5 and 10 days after salt stress, respectively.

Photosynthetic rate (*P*_n_) was significantly decreased in all three cultivars at the first 5 days of salt exposure, and this reduction was more pronounced at 10 days, as compared to CK (**Figure 5A**). The *P*_n_ of YZ9102 was found to a maximum reduction among three cultivars, decreased by 57.61% and 88.89% at 5 and 10 days under salt stress. The decrease of *P*_n_ in JHR1 reached to 33.13% and 73.76% after 5 and 10 days of salt stress, which was slightly higher than that in JHB1.

The stomatal conductance (*G*_s_) significantly decreased with the salt stress time (**Figure 5B**). The *G*_s_ in YZ9102 was highly decreased, followed by JHR1 and JHB1. In comparison with CK, the *G*_s_ significantly decreased by 73.95%, 44.35% and 36.71% in YZ9102, JHR1 and JHB1 at 5 days of salt stress, further it decreased by 85.95%, 67.78% and 63.95% at 10 days of salt stress, respectively.

The response patterns of transpiration rate (*T*_r_) in all three cultivars under salt stress was similar with that of their photosynthetic rate (*P*_n_) (**Figure 5C**). The maximum decrease in *T*_r_ was recorded in YZ9102 in all three cultivars, reaching 53.28% and 86.81% at 5 and 10 days of salt stress. The minimum decrease of *T*_r_ was recorded in JHR1 (40.04%) and JHB1 (74.20%), after 5 and 10 days of salt stress, respectively.

The variation of water use efficiency (*WUE*) in response to salt stress was observed in three cultivars (**Figure 5D**). The *WUE* of YZ9102 showed a slight decrease (9.08%) at 5 days under salt stress, and significantly decreased by 15.58% after 10 days as compared to CK. An increased trend in *WUE* was observed in JHR1 and JHB1 during 10 days of salt stress, which showed a stable increase in JHR1 at 11.5% approximately. The maximum increase (24.71%) of *WUE* was recorded at 5 days of salt stress in JHB1.

### 3.6 Chlorophyll fluorescence parameters

Salt stress significantly decreased photochemical efficiency (*F_v_*/*F_m_*), quantum yield of PS II (*ΦPSII*), electron transport rate (*ETR*) and photochemical quenching coefficient (*qP*) in all three cultivars (**Table 2**). The *F_v_*/*F_m_* was not significantly affected in all three cultivars at 5 days of salt stress, however it was significantly decreased after 10 days of salt stress as compared to CK. No significant difference was recorded for *F_v_*/*F_m_* between JHR1 and JHB1. However, *F_v_*/*F_m_* was highly decreased in the cultivar YZ9102. Similar trends were observed in *ΦPSII*, *ETR* and *qP* during 10 days under salt stress. The maximum decreases in *ΦPSII*, *ETR* and *qP* were observed in YZ9102, followed by JHR1 and JHB1 at the 10 days under salt stress as compared to CK.

**Table 2:** The chlorophyll fluorescence parameters of three peanut cultivars after 5 and 10 days under salt stress.

Days (d)	Cultivars	Treatment	*Fv*/*Fm*	*ΦPSII*	*ETR*	*qP*	*qNP*
5	YZ9102	CK	0.89 ± 0.02a	0.28 ± 0.02a	2.51 ± 0.21a	0.32 ± 0.01a	0.90 ± 0.10b
		SS	0.87 ± 0.02b	0.23 ± 0.02b	2.06 ± 0.17b	0.26 ± 0.02b	1.27 ± 0.13a
	JHR1	CK	0.89 ± 0.01a	0.27 ± 0.03a	2.50 ± 0.30a	0.29 ± 0.04a	0.95 ± 0.03a
		SS	0.88 ± 0.01a	0.25 ± 0.02a	2.34 ± 0.20a	0.27 ± 0.03a	1.00 ± 0.04a
	JHB1	CK	0.88 ± 0.01a	0.33 ± 0.03a	2.83 ± 0.31a	0.35 ± 0.03a	0.95 ± 0.09b
		SS	0.87 ± 0.02a	0.31 ± 0.03a	2.62 ± 0.24a	0.33 ± 0.03a	1.11 ± 0.13a
10	YZ9102	CK	0.93 ± 0.03a	0.33 ± 0.02a	2.69 ± 0.20a	0.37 ± 0.02a	0.87 ± 0.04b
		SS	0.86 ± 0.01b	0.24 ± 0.02b	1.91 ± 0.19b	0.27 ± 0.03b	1.15 ± 0.13a
	JHR1	CK	0.91 ± 0.01a	0.32 ± 0.03a	2.83 ± 0.25a	0.35 ± 0.04a	0.82 ± 0.03a
		SS	0.88 ± 0.0 b	0.25 ± 0.02b	2.23 ± 0.20b	0.29 ± 0.02b	0.84 ± 0.05a
	JHB1	CK	0.91 ± 0.01a	0.35 ± 0.03a	2.84 ± 0.20a	0.39 ± 0.03a	0.92 ± 0.05a
		SS	0.88 ± 0.02b	0.29 ± 0.02b	2.31 ± 0.12b	0.32 ± 0.03b	1.02 ± 0.08a

Data are means ± SD of five biological replicates. Different lowercase letters in the same column indicate significantly different between CK and SS at p<0.05. CK, meaning plants were watered by Hoagland solution without NaCl, and SS, meaning plants were watered by Hoagland solution with 150 mM NaCl.

The non-photochemical quenching coefficient (*qNP*) was significantly increased after salt exposure for 5 days as compared to CK, but that showed an inferior increase in three cultivars at 10 days under salt stress (**Table 2**). The *qNP* in YZ9102 increased by 40.03% and 31.88% after 5 and 10 days for salt stress, that exhibited the maximum increase in three cultivars. The *qNP* in JHB1 increased by 17.09% and 10.12% after 5 and 10 days for salt stress, as compared to CK. JHR1 showed the minimum increase in *qNP*.

## 4 Discussion

Peanut is moderately sensitive or comparatively sensitive (Meena et al., 2016) to soil saline stress conditions. Previous reports suggested that there is huge genetic diversity in the salt tolerance among peanut germplasms (Pal and Pal, 2022). The agronomic traits such as survival under salt stress, plant height, relative growth rate reflected the salt tolerance of peanut (Yasmine et al., 2019). The salt tolerance coefficient that was calculated as biomass accumulation ratio under salt stress to unstressed control in each genotype was considered as selection criteria for salt tolerance in peanut (Pal and Pal, 2022). In the present study, the maximum inhibition of growth and biomass under salt stress was recorded in YZ9102, followed by JHR1 and JHB1. Our results suggested that JHB1 is a relatively salt-tolerant cultivar compared to YZ9102. Higher levels of anthocyanin in cultivars showed stronger salt tolerance, as compare to the cultivars with low anthocyanin content which has been observed in rice (Chunthaburee et al., 2016) and *Brassica napus* (Kim et al., 2017).

The homeostasis of intracellular K^+^, Ca^2+^ and Na^+^ concentrations under salt stress is essential for maintaining membrane potential, and for the activities of many enzymes and an appropriate osmotic regulation (Zhu, 2003). Ion imbalances induced oxidative stress in response to imbalances in ROS, and resulted in nutrient deficiency and ion toxicity. In the current study, Na^+^ accumulation was significantly increased in leaves and stems under salt stress. However, a slight or significant increase in the content of K^+^ and Ca^2+^ under salt stress was observed in leaves of three peanut cultivars. This might suggest that peanut can improve relatively stable K^+^ and Ca^2+^ accumulation in leaves by adjusting the transport capacity of mineral ions to alleviate the adverse effect of Na^+^ excessive accumulation (Li Q. et al., 2022). In our study, the K^+^/Na^+^ and Ca^2+^/Na^+^ were significantly decreased in all three cultivars under salt stress, and decreased more severely in stems than that in leaves. Similar results have been observed in wheat (Rahnama et al., 2011) and rice (Nemati et al., 2011). This might suggest that the selective ion transport and partitioning may be contributing to the adaptation to salt stress of plants (Ran et al., 2021). In this study, the descend range of K^+^/Na^+^ and Ca^2+^/Na^+^ were similar in leaves or stems among the three cultivars. Our results are inconsistent with the previous reports suggesting that the ion ratio was related to salt tolerance under salt stress (Guo et al., 2022). These results indicated that the salt tolerance of black peanut may not be related to ion absorption.

Flavonoid is an important plant secondary metabolite, is proved to be major component of plant antioxidant defense system against abiotic stresses through preventing generation of ROS or scavenging already generated ROS (Hernández et al., 2009). In the present study, salt stress significantly increased the contents of flavonoid and anthocyanin in all three cultivars. Similar results have been reported in *Brassica napus* (Kim et al., 2017) and pea (Farooq et al., 2021), which found that the contents of total flavonoid and phenolic compounds showed a significant increase to improve resistance in response to salt stress. In this study, JHR1 and JHB1 showed the higher increase of anthocyanin and flavonoid than YZ9102 under salt stress. These findings are consistent with wheat (Liu et al., 2012), suggesting that the salt-tolerant cultivars had higher increase of anthocyanin content than salt-sensitive under salt stress. With or without salt stress, the anthocyanin and flavonoid contents of JHB1 were higher than those of JHR1 and YZ9102, suggesting that the salt-tolerant cultivars had higher anthocyanin content than the salt-sensitive cultivars to possess more physiological activities (Daiponmak et al., 2010). Majority of the research community has confirmed these results. Over expression the genes related to the flavonoid and anthocyanin biosynthesis in transgenic plants increased the flavonoid and anthocyanin accumulation, enhanced the oxidation resistance and salt tolerance (Li et al., 2016; Wang et al., 2016).The anthocyanin free (Kang et al., 2014) and flavonoid deficiency (Sugimoto et al., 2021) mutants failed to produce anthocyanin and flavonoid in all tissues because of inactivation of flavonoid biosynthetic enzymes, and increased production of reactive oxygen species (ROS).

High concentration of salt stress caused rapid increase of ROS, which could perturb cellular redox homeostasis, results in oxidative stress and induce a series of cell damage. The ROS scavenging system of plants could be activated to alleviate such oxidative damages for enhancing salt tolerance (Choudhury et al., 2013). The antioxidative enzymes including SOD, POD and CAT could cooperatively scavenge the ROS and maintain the ROS below toxic range (Kaya et al., 2020). In this study, the antioxidative enzyme activities of SOD, POD and CAT were activated by salt stress in JHR1 and JHB1, however, those were restrained in YZ9102. Different responses of enzyme activities to salt stress may be responsible for the different sensitivities among the cultivars under salt stress (Guo et al., 2022). The activation of enzyme activities was more obvious in JHB1 than JHR1. Similar with our findings, cultivars e.g. maize (Hichem et al., 2009) and apple (Wang et al., 2015) with more intense anthocyanin showed higher increase of total antioxidant activity than less ones under salt stress, and exhibited more redox stabilization and better behavior of salt-challenge. Exogenous anthocyanin treatments suggested that anthocyanin not only acted as free radical scavengers but promoted activation of antioxidant enzymes and other non-enzymatic antioxidants, and improved the physiological state of plants (Paul et al., 2017; Maleva et al., 2018), which indirectly confirmed our results. In the current study, the activation effect of three antioxidant enzyme activities in JHB1 gradually increased from 1 to 10 d after salt stress, however those in JHR1 were found to decrease or inactivate at late stage compared with the early salt stress. It may be attributed to that there were differences with resistance to salt stress duration and intensity among cultivars (Wang et al., 2019; El-Badri et al., 2021). In our study, MDA content showed a maximum and minimum increase in YZ9102 and JHB1 under salt stress, respectively. Similar results in maize (Hichem et al., 2009) also suggested that genotypes with high anthocyanin content were able to maintain lower MDA content and significant higher dry matter production than yellow ones upon salt stress. The MDA content in YZ9102 showed a rapid increase, however, it was slowly increased in JHB1 under salt stress. The result was consistent with the trend of enzyme activities of three cultivars. Our results indicated that activating antioxidant enzymes via anthocyanin under salt stress could effectively alleviate cell damage caused by oxidative stress (Daiponmak et al., 2010).

Photosynthesis reduction under salt stress is mainly due to stomatal closure and CO_2_ diffusion hindered via a reduction in guard cell turgor, and partially to photosystem II (PSII) photo inhibition. The net photosynthetic rate decreased with increasing intensity of salt stress, and showed greater reduction in the salt-sensitive cultivar than that in tolerant (Dionisio-Sese and Tobita, 2000). Similar results were obtained in this study. The photosynthetic traits (*P*_n_, *G*_s_, and *T*_r_) significantly decreased at 5 days under salt stress, and further decreased at 10 days of salt stress. Maximum reductions of photosynthetic traits were recorded in cultivar YZ9102, followed by JHR1 and JHB1. It may be attributed to that the tolerant cultivars had more responsive stomata that tended to close faster when exposure to salt stress at the first few hours, followed by partial recovery after a temporary acclimation. However, the recovery ability of stomata in sensitive cultivars was deficiency coping with salt stress (Foad and Ismail, 2007). Similar results in water spinach also suggested that *P*_n_, *G*_s_, and *T*_r_ in green cultivar were more sensitive to salt stress than that in red cultivar, and showed a severe decline under the same salt stress (Kitayama et al., 2019). Exogenous antioxidant alleviated photosynthesis inhibition in rapeseed (Ma et al., 2017) and soybean (Alharbi et al., 2021) under salt stress by activating antioxidant systems, mitigated the salt stress damage, which explained that the photosynthetic capacity in more intense in anthocyanin rich cultivars were less affected by salt stress. The water use efficiency (WUE), estimated as the ratio of *P*_n_ to *T*_r_, showed a gradually downward in tomato when exposure to 0.3%~0.9% salt stress (Yang et al., 2019). However, WUE exhibited a significant increase in tartary buckwheat under 100 mM NaCl stress, and a little change in common buckwheat (Matsuura et al., 2005). The response of WUE to salt stress represented the salt tolerance of different species or cultivars. In this study, the WUE was found to a downward in YZ9102 during salt stress duration, and showed an increase trend in JHR1 and JHB1after10 days of salt stress. It may be mainly attributed to the diverse maintenance of *P*_n_ and *G*_s_ under salt stress.

Chlorophyll fluorescence, providing insights into the response of photosynthesis to environmental stresses, is a rapid and non-destructive tool used to screen cultivars for salt tolerance (Zribi et al., 2009). In the present study, the chlorophyll fluorescence including *F_v_*/*F_m_*, *ΦPSII*, *ETR* and *qP* showed a slight reduction in three cultivars at 5 days after salt stress, and significantly decreased at 10 days. Similar result was found in sorghum (Netondo et al., 2004). It may be responsible for the PSII photo inhibition turn into the main limitation of photosynthesis reduction at late stage of salt stress (Dionisio-Sese and Tobita, 2000). The maximum decrease in chlorophyll fluorescence was observed in YZ9102, followed by JHR1 and JHB1 under salt stress. Similar result in brassicas suggested that salt-tolerant cultivars exhibited better PSII quantum efficiency and utilization of photochemical energy than salt-sensitive cultivars under salt stress (Ahmed et al., 2023). Water spinach cultivars with higher anthocyanin content also showed lesser inhibition in PSII quantum efficiency than those with lower anthocyanin content (Kitayama et al., 2019). In this study, the *qNP* in YZ9102 showed the maximum increase under salt stress, followed by JHB1 and JHR1. Our results are consistent with rice (Foad and Ismail, 2007), suggesting that PSII reaction center could alleviate excitation pressure via diverting light energy into heat to maintain an adequate balance between absorption and utilization of light (Krause and Weis, 1991). Salt stress inhibited electron transport involving PSII, and increased the *qNP* in Arabidopsis, however, did not affected the photochemical efficiency in *Thellungiella* (Stepien and Johnson, 2009), suggesting that the photosystem of salt-tolerant crops showed greater stability.

## 5 Conclusions

In conclusion, a prominent difference in response to salt stress among the three tested cultivars of different testa color was observed in this study. The strength of salt tolerance was higher in black color testa genotype (JHB1) followed by red (JHR1) and pink (YZ9102) on the basis of the relative growth value. JHB1 showed maximum accumulation of flavonoid and anthocyanin than JHR1 and YZ9102 with or without salt stress. In salt treated, the ion imbalances, expressed as the ratio of K^+^/Na^+^ and Ca^2+^/Na^+^, were shown to be similar among the three cultivars. The relative activation of antioxidant enzyme activities and membrane stability in JHB1 were more outstanding than the other two cultivars when subjected to salt stress. The most tolerant to salt stress in JHB1 was mainly attributed to the accumulation of the anthocyanin and flavonoid activating antioxidant protection against the oxidative damage to maintain the higher photosynthetic efficiency and plant growth under salt stress. It is necessary to study the underlying molecular mechanisms of salt tolerance in black peanut or exogenous anthocyanin regulated salt tolerance.

## Data availability statement

The original contributions presented in the study are included in the article/supplementary material. Further inquiries can be directed to the corresponding author.

## Author contributions

GL: Conceptualization, Formal analysis, Investigation, Methodology, Supervision, Writing – original draft, Writing – review & editing. XG: Data curation, Investigation, Writing – original draft. YS: Data curation, Investigation, Writing – original draft. SG: Writing – review & editing. KZ: Data curation, Methodology, Software, Writing – original draft. FW: Data curation, Investigation, Writing – original draft. GW: Data curation, Methodology, Software, Writing – original draft. HZ: Data curation, Investigation, Writing – original draft. AL: Data curation, Formal Analysis, Writing – original draft. XW: Funding acquisition, Project administration, Supervision, Writing – review & editing. CZ: Funding acquisition, Project administration, Supervision, Writing – review & editing.
